# Icons: identifying continence options after stroke trial: utility of a logic model in the design and implementation of a process evaluation

**DOI:** 10.1186/1745-6215-14-S1-O90

**Published:** 2013-11-29

**Authors:** Lois Thomas, Christopher Burton, Beverley French, Michael Leathley, Denise Forshaw, Christopher Sutton, Brenda Roe, Brigit Chesworth, Caroline Watkins

**Affiliations:** 1 University of Central Lancashire, Preston, UK; 2 Bangor University, Bangor, UK; 3 Edge Hill University, Ormskirk, UK

## Background

ICONS is a cluster randomised controlled pilot trial designed to provide preliminary evidence of the effectiveness of a systematic voiding programme (SVP) for the management of continence after stroke. Stroke services were randomised to receive the SVP, the SVP plus supported implementation, or usual care. Process evaluations are designed to evaluate fidelity and provide explanatory evidence around trial outcomes; these need to be underpinned by a theoretical framework to explain linkages between intervention processes and outcomes.

## Process evaluation

We conducted an evaluation to describe SVP implementation and assist in explaining intervention outcomes. Reflecting best practice in complex intervention research, we developed a logic model to underpin the evaluation representing practitioners' implementation activities. To increase explanatory power of the model, we synthesised principles from theoretical frameworks underpinning the study (e.g. the Normalisation Process Model) into mechanisms of action to explain conditions necessary for activities to impact on outcomes. Mechanisms were:

• **Understanding and agreeing:** conceptual work associated with the SVP, e.g. increasing awareness.

• **Driving and aligning:** organising systems or processes to align and drive new practice.

• **Building and supporting:** enacting the SVP.

• **Learning and evaluation:** reflecting on performance and progress.

## Findings

We will discuss the utility of the logic model in explaining conditions necessary for the intervention to work, the success of implementation strategies adopted and variations in patient outcome across trial arms. We will also consider the challenges of synthesising across multiple data sources to understand variation in intervention delivery, maintenance and outcome in cluster trials.

